# Design Optimization of Structural Parameters for Highly Sensitive Photonic Crystal Label-Free Biosensors

**DOI:** 10.3390/s130303232

**Published:** 2013-03-07

**Authors:** Jonghyun Ju, Yun-ah Han, Seok-min Kim

**Affiliations:** School of Mechanical Engineering, Chung-Ang University, 84 Heukseok-Ro, Dongjak-Gu, Seoul 156-756, Korea; E-Mails: jhju@cau.ac.kr (J.J.); han6970@cau.ac.kr (Y.H.)

**Keywords:** photonic crystal, guided mode resonance, biosensor, design optimization, desirability function

## Abstract

The effects of structural design parameters on the performance of nano-replicated photonic crystal (PC) label-free biosensors were examined by the analysis of simulated reflection spectra of PC structures. The grating pitch, duty, scaled grating height and scaled TiO_2_ layer thickness were selected as the design factors to optimize the PC structure. The peak wavelength value (PWV), full width at half maximum of the peak, figure of merit for the bulk and surface sensitivities, and surface/bulk sensitivity ratio were also selected as the responses to optimize the PC label-free biosensor performance. A parametric study showed that the grating pitch was the dominant factor for PWV, and that it had low interaction effects with other scaled design factors. Therefore, we can isolate the effect of grating pitch using scaled design factors. For the design of PC-label free biosensor, one should consider that: (1) the PWV can be measured by the reflection peak measurement instruments, (2) the grating pitch and duty can be manufactured using conventional lithography systems, and (3) the optimum design is less sensitive to the grating height and TiO_2_ layer thickness variations in the fabrication process. In this paper, we suggested a design guide for highly sensitive PC biosensor in which one select the grating pitch and duty based on the limitations of the lithography and measurement system, and conduct a multi objective optimization of the grating height and TiO_2_ layer thickness for maximizing performance and minimizing the influence of parameter variation. Through multi-objective optimization of a PC structure with a fixed grating height of 550 nm and a duty of 50%, we obtained a surface FOM of 66.18 RIU^−1^ and an S/B ratio of 34.8%, with a grating height of 117 nm and TiO_2_ height of 210 nm.

## 1. Introduction

A photonic crystal (PC) is a periodic arrangement of dielectric materials. PCs can support guided-mode resonance when the evanescent (cut-off) diffracted orders of a periodic sub-wavelength surface structure couple with the modes of an effective high-index layer. The energy is coupled with ‘leaky modes’ that escape from the structure in both the forward and backward directions because of its diffractive nature. They interfere with the directly transmitted and reflected zeroth orders: this leads to a strong reflection about a resonant wavelength whose line width and spectral location are set by the physical parameters of the device [1]. The resonance wavelength is very sensitive to changes in the refractive index (RI) of the materials surrounding the PC. Thus, the PC structure can be used as a biosensor that detects the change in the surrounding RI due to biomolecular interactions [2]. Furthermore, a PC label-free biosensor can be fabricated by inexpensive nano-replication and physical vapor deposition processes. PC biosensors eliminate the need for complex measurement instruments and have high spatial resolution. Therefore, there is a growing interest in PC label-free biosensors for drug discovery, medical diagnostics, and life-science research [3].

As with other sensors, the detection sensitivity is the critical factor in PC label-free biosensors. Some approaches proposed to improve their detection sensitivity include the following: Ganesh *et al.* [4] suggested a near-ultraviolet wavelength PC biosensor to enhance the surface-to-bulk sensitivity ratio. Zhang *et al.* [5] proposed a porous nanorod structure to improve the surface area and detection sensitivity. Block *et al.* proposed a highly sensitive PC biosensor using a low index sol-gel glass nanograting [6], and a sensitivity model that showed how bulk and surface sensitivities are related to the spatial electromagnetic field distribution of the PC [7]. Although various approaches for improving the sensitivity of PC label-free biosensors by changing the structural parameters or material properties have also been proposed, a full parametric study of the structural design factors on the performance of PC label-free biosensor has not been conducted, which is important to understand the nature of PC label-free biosensor. Furthermore, an optimization considering the interaction effects of design factors should be conducted for maximizing the sensor performance, because the global optimum of PC structure cannot be obtained by a one-factor-at-a-time optimization method due to the very non-linear characteristic (strong interaction effects of design factors) of PC optical resonator. Here, we propose a methodology for examining the detection sensitivity of PC label-free biosensors, and examined the influence of each structural factor on the responses using rigorous coupled wave analysis (RCWA). We also applied an optimization method that considers the interaction effects and limits of fabrication/readout instruments as a design guideline for PC label-free biosensors.

## 2. Selection of Design Factors and Responses

**Figure 1.**
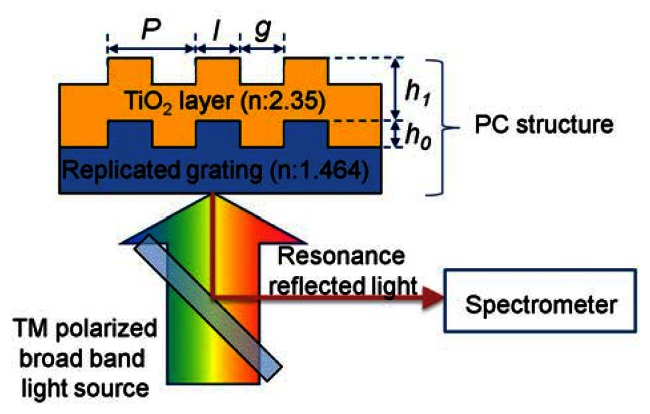
Schematic diagram of a PC structure composed of a replicated grating and a TiO_2_ layer. The method for detecting the resonance reflection peak is also shown.

Among the various structures and materials, a PC composed of a nano-replicated polymer grating (*n* = 1.464) and a TiO_2_ high-index layer (*n* = 2.35), as depicted in Figure 1, was used for the design target [1]. The grating pitch *P*, land width *l*, replicated grating height, and thickness of the TiO_2_ layer were selected as the design factors. Since the optimization process considering full design factors is time consuming and may provide meaninglessness results (the optimum structure is too hard to fabricate or the performance of the PC structure is too hard to measure), the scaled design factors were used to provide an effective and reasonable design guideline for PC biosensor. The duty (*l*/*P*), and scaled grating height *h*_0_ and TiO_2_ height *h*_1_ relative to the grating pitch *P* were used for parametric study of the effect of design factors on the performance of PC biosensors, because the scaling factors showed similar performance trends for the entire range of the grating pitch, and the effect of grating pitch can be isolated using the scaled other design factors. A *P* of 250–550 nm, *D* of 0.2–0.8, and *h*_0_ and *h*_1_ of 0.1–0.5 were used in this study. The range of design parameters was selected considering the limits of inexpensive nano-pattering processes and readout instruments.

To represent the performance of PC label-free biosensors, the following responses were selected: the peak wavelength value (PWV), the full width at half maximum (FWHM) of the peak, the ‘figure of merit’ (FOM), and the surface to bulk ratio (S/B ratio). The PWV is an important design factor for PC label-free biosensors, because the target PWV range can depend on the readout instruments. In addition, some bio-molecules can be damaged by illumination at a specific wavelength of light. The FWHM of the resonance peak is also important for defining the performance of optical resonance sensors, because the sensor resolution is inversely related to the FWHM [8]. To compare the performance of optical resonance based sensors, the FOM was defined as [9]:

(1) FOM=Sensitivity(nm/RIU)/FWHM(nm).

and applied to represent the characteristics of PC label-free biosensors. In Equation (1), the sensitivity is the slope of the PWV with respect to the RI. The sensitivity of PC label-free biosensors can be described in terms of the surface and bulk sensitivities. The surface sensitivity is useful in the context of detecting thin layers of adsorbed biomolecules, and the bulk sensitivity is correlated with the detection of larger objects such as cells or RI fluctuations of the test media. Because variations in the bulk solution RI are a significant source of noise, higher surface sensitivity and lower bulk sensitivity are preferred for surface biosensors. Therefore, the S/B ratio was selected as a response that is important for surface biosensors.

**Figure 2.**
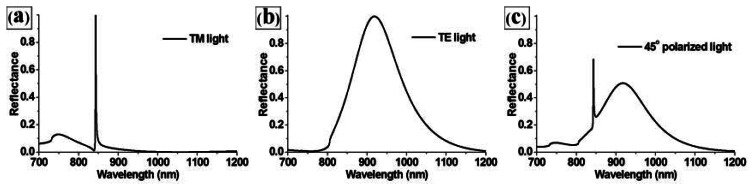
Comparison of the reflection peak spectra of the PC structure (*P* = 550 nm, *D* = 0.5, *h_0_* = 0.3, and *h_1_* = 0.2) for (**a**) TM, (**b**) TE, and (**c**) 45-degree polarized light sources.

An RCWA (DiffractMod, RSOFT) study was performed to calculate the responses, because it could estimate the performance of PC structures quite closely [6]. Since the resonance characteristics of PC structures are sensitive to the polarization direction, we compared the simulated reflection spectra of the PC structure (*P* = 550 nm, *D* = 0.5, *h*_0_ = 0.3, and *h*_1_ = 0.2) in water solution for the light sources with different polarizations as depicted in Figure 2. The reflection spectrum obtained with transverse magnetic (TM)-polarized light at normal incidence (where the electric field was perpendicular to the grating lines) shows a sharper reflection peak than transverse electric (TE)-polarized light (where the electric field is parallel to the grating lines). In addition, 45-degree polarized light shows a superposition of reflection peaks for TE and TM light sources, and it may confuse the detection of peak changes because of the biomolecular interaction. Therefore, we selected TM light sources for highly sensitive PC biosensor.

To calculate the surface sensitivity, the surface region was assumed to be a volume lying within 25 nm from all exposed surfaces at the top of the PC. The sample materials in the PC biosensor were assumed to have a RI range of 1.2 to 1.5 (*RI_surf_*), and water (*n* = 1.33) was used as a bulk solution. To calculate the bulk sensitivity, the RI of the bulk solution (*RI_bulk_*) was assumed to be between that of water (*n* = 1.33) and isopropyl alcohol (IPA, *n* = 1.38).

## 3. Parametric Study of Design Factors

Because the response of the PC to normally incident illumination is coupled with the second-order Bragg condition, the PWV is proportional to the grating pitch and RI such that:

(2) $$\text{PWV}=n_{\text{eff}}P$$

**Figure 3.**
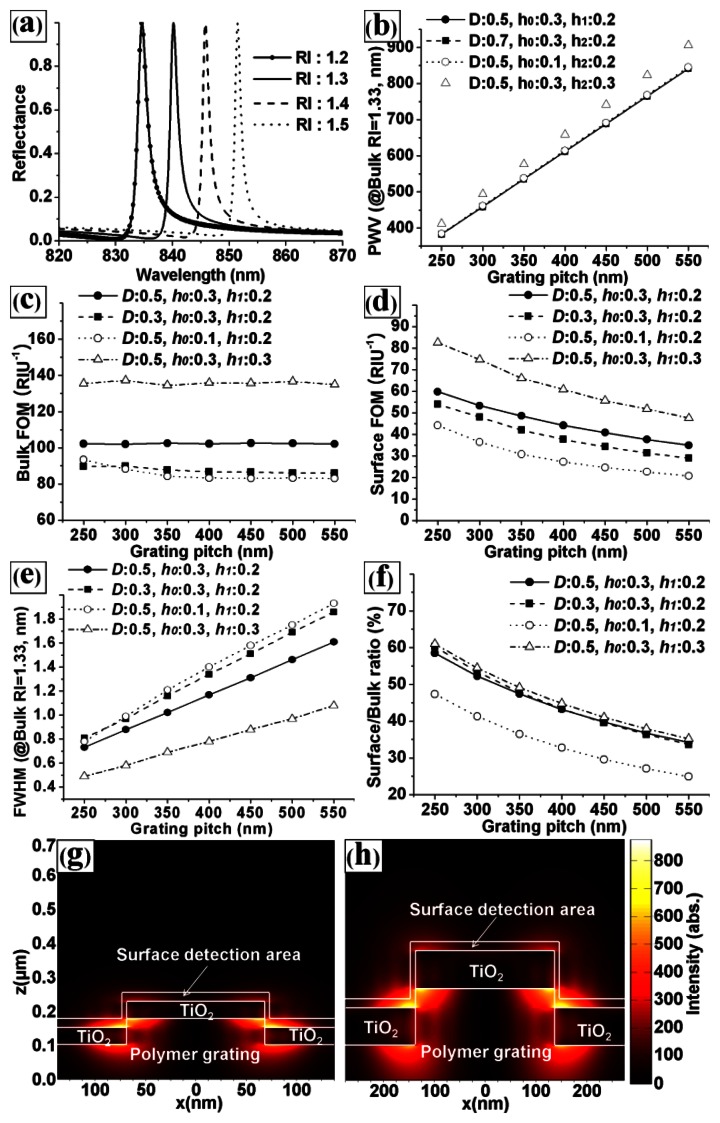
(**a**) Simulated reflection spectra of the PC structure for various sample refractive indexes at fixed design factors (*P* = 550 nm, *D* = 0.5, *h_0_* = 0.3, and *h_1_* = 0.2). (**b**–**f**) Effects of grating pitches for four PC structures with different other design factors on: the PWV at a sample RI of 1.33, the bulk FOM, the surface FOM, the FWHM, and the surface/bulk ratio (S/B ratio), respectively. (The base structure was *D* = 0.5, *h_0_* = 0.3, and *h_1_* = 0.2). (**g,h**) Electric field intensity distribution on the PC structure for grating pitches of 250 and 550 nm, respectively, under fixed design factors (*D* = 0.5, *h_0_* = 0.3, and *h_1_* = 0.2).

In Equation (2)*n_eff_* is the effective RI of the medium [7]. Figure 3(a) shows the simulated reflection spectra of the PC structure for various values of *RI_surf_* at fixed design factors (*P* = 550 nm, *D* = 0.5, *h*_0_ = 0.3, *h*_1_ = 0.2, and *RI_bulk_* = 1.33). The graph shows that the PWV was proportional to the *RI_surf_*, as expected from Equation (2). Hence, the PC can be used as a sensor for detecting changes in the *RI_surf_*. Figure 3(b–f) shows the effects of the grating pitch on the responses representing the performance of PC biosensor. The effects of grating pitch for the base structure (*D* = 0.5, *h_0_* = 0.3, and *h_1_* = 0.2, circle dot line) and the other structures in which one design factor was changed from the base structure; *D* was changed in square dot line, *h_0_* was changed in open circle line, and *h_1_* was changed in open square line. Figure 3(b–f) clearly shows the effects of grating pitch on the responses and that there are weak interaction effects between grating pitch and the other scaled design factors (the graphs are almost parallel to each other). It is clear evidence that the effects of grating pitch can be isolated using scaled other design factors. Figure 3(b) shows the effect of grating pitch on the PWV of the PC. The PWV increased with the grating pitch for four specific PC designs and PCs with different *h*_1_ values showed different results. It was noted that *P* and *h*_1_ were dominant factors for PWV. Figure 3(c,d) shows the effects of the pitch on the surface FOM (surface sensitivity / FWHM) and bulk FOM (bulk sensitivity / FWHM). The bulk FOM was independent of the grating pitch. However, the surface FOM decreased as the grating pitch increased. These results can be explained by the effective RI model [7] and the relationship between the FWHM and the pitch (shown in Figure 3(e)). The change in the effective RI (*n_eff_*) in Equation (2) was affected by the integrated product of the change in the dielectric permittivity and the electric-field intensity in the region of detection.

Figure 3(g,h) shows the simulated electric-field intensity distributions for grating pitches of Figure 3(g) 250 and Figure 3(h) 550 nm at the resonance condition. The ratios of the integrated electric field intensity of the surface detection area to that of the entire PC top area for pitches of 250 and 550 nm were Figure 3(g) 62.99% and Figure 3(h) 37.24%, respectively. Clearly, the ratio of the integrated electric-field intensity for the surface region increased as the grating pitch decreased. Hence, the surface sensitivity was unaffected by the grating pitch, because Δ*n_eff_* decreases and P increases in Equation (2) as the grating pitch increases. Because the FWHM is proportional to the pitch, however, the surface FOM increased as the pitch decreased. In the case of the bulk FOM, the bulk sensitivity increased with the grating pitch, while the bulk Δ*n_eff_* was almost independent. The FWHM also increased as the grating pitch increased. Therefore, the bulk FOM was independent of the grating pitch, and the S/B ratio increased with the grating pitch, as depicted in Figure 3(f). The results also show that the bulk FOM, surface FOM, FWHM, and S/B ratio were also affected by the duty, *h*_0_, and *h*_1_. Although Figure 3(b–f) shows the effects of the grating pitch for four specific PC designs, we conducted a similar analysis for various PC designs and obtained similar trends. Because we used scaled design factors (except for the grating pitch), the grating pitch did not interact strongly with other design factors. Hence, the fine-tuning of the other design factors is more important in the design process of highly sensitive PC biosensors.

**Figure 4.**
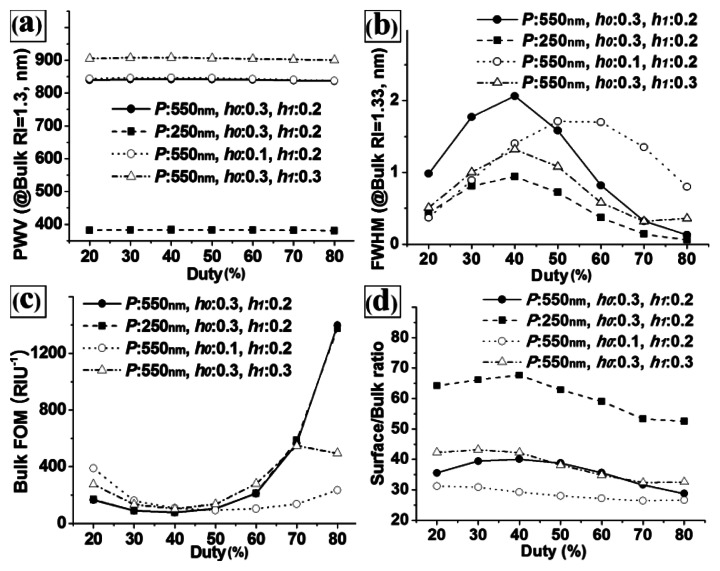
Effects of the duty on the (**a**) PWV, (**b**) FWHM, (**c**) bulk FOM, and (**d**) S/B ratio for four different PC structures (base structure: *P* = 550 nm, *h*_0_ = 0.3, and *h*_1_ = 0.2).

Figure 4 shows the effects of the duty on the calculated (a) PWV, (b) FWHM, (c) bulk FOM, and (d) S/B ratio responses for the base structure (*P* = 550 nm, *h*_0_ = 0.3, and *h*_1_ = 0.2) and the other structures in which one design factor was changed from the base structure. As with the results in Figure 3, the duty strongly affected the FWHM and FOM, but not the PWV and S/B ratio. It is also noted that the trends for the FWHM, FOM, and S/B ratio with regard to the duty were similar across the PC structures with different pitches (see the black circle dot graph (*P* = 550 nm) and the black square dot graph (*P* = 250 nm)), because we used scaled design factors. However, the trends were not similar between structures with different values of *h*_0_ and *h*_1_. This revealed that there were strong interaction effects between *D*, *h*_0_, and *h*_1_. Therefore, optimization of multiple design factors considering the interaction effects is required to maximize the performance of PC label-free biosensors.

## 4. Optimization of the Scaled Grating Height and TiO_2_ Thickness

Because we used scaled factors for the duty, grating height, and thickness of the TiO_2_ layer, the grating pitch did not show any interaction effects with the other design factors. Therefore, we were able to isolate the pitch for the optimization of PC-label free biosensors. Regarding the remaining three design factors, we could fix the duty depending on limitations in the fabrication process. Highly expensive patterning equipment cannot be used to pattern the master of PC label-free biosensors to match their disposable characteristics. Considering the critical dimension of the KrF laser projection lithographic system that is generally used to fabricate PC masters (∼130 nm), a duty of 50% is preferable for most PC label-free biosensors. Although a duty of ∼50% showed relatively large FWHM in Figure 4, the manufacturability is more important factor for real biosensor application. Duties of 30–70% can also be used for PCs with large grating pitches. For the design of highly sensitive PC biosensors, a selection of grating pitches and duties should be determined considering the lithography system and target PWV range, and an optimization of *h*_0_ and *h*_1_ should be conducted. Because optimization is a way to not only find structural parameters that maximize the performance of the device but also minimize the effects of parameter variation, the optimization process of *h*_0_ and *h*_1_ is rational, since the dimensional accuracies and repeatability of the grating height and TiO_2_ layer thickness during fabrication are lower than those of the grating pitch and duty.

**Figure 5.**
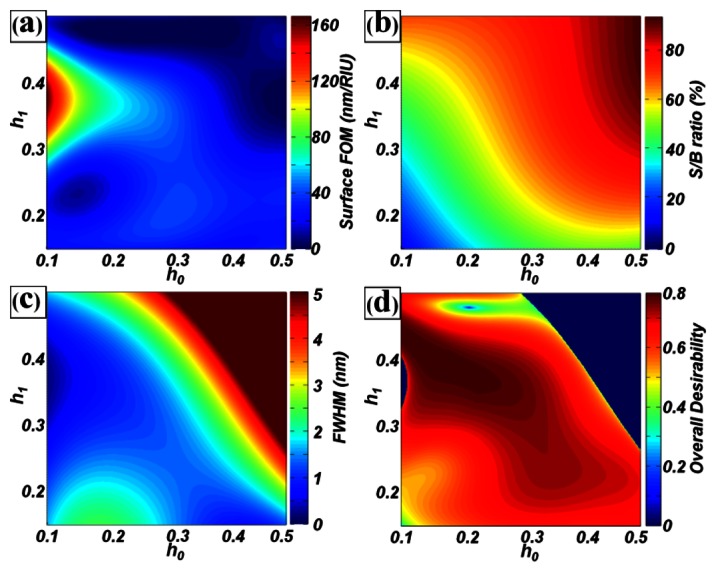
Response surfaces of the (**a**) surface FOM, (**b**) S/B ratio, (**c**) FWHM, and (**d**) overall desirability for the design factors of *h*_0_ and *h*_1_ (*P* = 550 nm and *D* = 0.5).

In this study, as an example, design optimization was undertaken for PC label-free biosensors with a pitch of 550 nm and duty of 50%, to minimize the detection limits of surface biosensors. For this purpose, the surface FOM (*y*_1_) and S/B ratio (*y*_2_) were selected as the objective functions. Because spectral peaks with very narrow bandwidth require an expensive spectrum analyzing system, the FWHM (*y*_3_) was also considered as the objective function. To optimize the two design factors and three responses, a factorial design of experiments was first performed for *h*_0_ and *h*_1_. The data were then fitted using an *n*^th^ order polynomial regression function based on the least-squares method:

(3) $$\begin{array}{ll} y_{k}=\sum_{i=0}^{n}\sum_{j=0}^{i}a_{ij}h_{0}^{\left(i-j\right)}h_{1}^{j} & k=1,2,3 \end{array}$$

where *a_ij_* is the coefficient and *y_k_* is the k^th^ estimate response. To obtain the estimate responses with a coefficient of determination *r^2^* of more than 95%, a sixth-order polynomial function was used for the surface FOM (*y*_1_) and a third-order function was used for both the S/B ratio (*y*_2_) and FWHM (*y*_3_). Figure 5(a–c) shows the response surface for the (a) surface FOM, (b) S/B ratio and (c) FWHM. Because there were three independent objectives for the different dimensions and orders of magnitude to optimize, a desirability function [10] was used. For the surface biosensor, “the larger the better” rule applied for the surface FOM (*y*_1_) and S/B ratio (*y*_2_). These parameters could be transformed to *d*_1_ and *d*_2_ as:

(4) $$d_{i}=\{\begin{array}{ccc} 0 & y_{i}<L_{i}\\ {\left(\frac{y_{i}-L_{i}}{U_{i}-L_{i}}\right)}^{q_{i}} & L_{i}\leq y_{i}\leq U_{i} & i=1,2\\ 1 & U_{i}<y_{i} \end{array}$$

where *L_i_* is the lower limit and *U_i_* is the upper limit of *y_i_* in the experimental region, and *q_i_* is a weight factor which has temporary values. The FWHM characteristic was assigned as “nominal is better”, because the designers of PC biosensors can have a preferred FWHM, depending on the readout system. Hence, the FWHM (*y*_3_) was transformed to *d*_3_ as:

(5) $$d_{3}=\{\begin{array}{cc} 0 & y_{i}<L_{3}\\ {\left(\frac{y_{3}-L_{3}}{T-L_{3}}\right)}^{q_{3}} & L_{3}\leq y_{i}\leq T_{3}\\ {\left(\frac{U_{3}-y_{3}}{U_{3}-T}\right)}^{q_{4}} & T_{3}\leq y_{i}\leq U_{3}\\ 0 & U_{3}<y_{i} \end{array}$$

where *T* is the target value of the FWHM and *L_3_* and *U_3_* were the lower and upper limits. In this study, the target value, lower limit, and upper limit of the FWHM were set 1, 0.3, and 5 nm, respectively. The lower limit was selected considering the spectral resolution of conventional compact spectroscopy systems. Because *d*_1_ and *d*_2_ are more important than *d*_3_ for highly sensitive PC biosensors, the weighting factors *q*_1_, *q*_2_, *q*_3_, and *q*_4_ were set to be 0.5, 0.5, 0.2, and 0.2, respectively. After estimate responses were transformed into individual desirability functions *d_i_* (0 < *d_i_* < 1), the overall desirability function *F* could be calculated as:

(6) $$\text{F}={\left({\text{d}}_{1}\times {\text{d}}_{2}\times {\text{d}}_{3}\right)}^{1/3}$$

The overall desirability function *F* is shown in Figure 5(d). The maximum value of *F* was 0.7838 and the (*h*_0_, *h*_1_) values were (0.2135, 0.3816). These scaled values were transformed into real values of the structural dimensions, such as a grating height of 117 nm and TiO_2_ layer thickness of 210 nm. Under these conditions, the estimated surface FOM (*y*_1_) was 62.73 RIU^−1^, the S/B ratio (*y*_2_) was 34.80%, and the FWHM (*y*_3_) was 0.78 nm. From the RCWA simulation of PC label-free biosensors with optimal structures, the calculated surface FOM was 66.18 RIU^−1^, the S/B ratio was 34.72%, and the FWHM was 0.62 nm. We noted that the estimated values were similar to the calculated values. This clearly revealed that our design approach was reasonable. Compared to the base model (*P* = 550 nm, *D* = 0.5, *h*_0_ = 0.3, *h*_1_ = 0.2), the surface FOM was improved from 34.90 to 66.18 RIU^−1^ (∼ 90% enhancement), while the S/B ratio was maintained (34.2 and 34.8% for the base and optimal models) in the optimal design.

## 5. Conclusions

We performed a parametric study of the effects of structural design factors on the performance of PC label-free biosensors. The bulk and surface FOM, S/B ratio, and FWHM were proposed to define the performance of PC biosensors. To minimize the interaction effects and isolate the effect of grating height, the scaled design factors to the grating pitch were used in the parametric study. From the parametric study, the duty, scaled grating height, and scaled TiO_2_ thickness were found to have only slight interactions with the grating pitch but considerable interactions with each other in terms of the PC biosensor performance. Because the grating pitch and duty are difficult to control because of limitations in lithography systems (unlike the grating height and TiO_2_ thickness), we proposed a design rule for highly sensitive PC biosensors in which a grating height and duty are first selected by considering the lithography system capability and target PWV value, and then a multi-objective optimization process for the grating height and TiO_2_ thickness is considered. Because of the relatively lower dimensional accuracy and repeatability of the grating height and TiO_2_ layer thickness during fabrication compared to the grating pitch and duty, the proposed optimization method is useful for minimizing the performance variations caused by changes in the former variables. In this study, a surface FOM of 66.18 RIU^−1^ and S/B ratio of 34.8% was achieved for an optimal surface biosensor structure with a pitch of 550 nm, a duty of 0.5, a grating height of 117 nm, and TiO_2_ layer thickness of 210 nm. The optimized structural values of the scaled grating height and TiO_2_ layer thickness could not be applied to PC structures with different grating pitches and duty, because the FWHM was affected by the grating pitch and the duty showed strong interaction effects with *h*_0_ and *h*_1_. However, we believe that the results of this work will be valuable for understanding the influence of the design parameters on PC biosensor performance, and for designing highly sensitive and robust PC biosensors. Furthermore, the results are invaluable for the optimization of other PC applications, including (but not limited to) optical filters, PC SERS biosensors, and enhanced-fluorescence biosensors.
